# Asthma and pregnancy

**DOI:** 10.61622/rbgo/2025FPS5

**Published:** 2025-07-02

**Authors:** Renato Teixeira Souza, Inessa Beraldo de Andrade Bonomi, Carlos Alberto Maganha, Elton Carlos Ferreira, Sara Tossa Gomes Solha, Janete Vetorazzi, Rosiane Mattar, Regina Maria de Carvalho-Pinto, Thiago Prudente Bártholo, Lilian Serrasqueiro Ballini Caetano

**Affiliations:** Universidade Estadual de Campinas Campinas SP Brazil Universidade Estadual de Campinas, Campinas, SP, Brazil.; Universidade Professor Edson Antônio Velano Belo Horizonte MG Brazil Universidade Professor Edson Antônio Velano, Curso de Medicina, Belo Horizonte, MG, Brazil.; Faculdade de Ciências Médicas de São José dos Campos São José dos Campos SP Brazil Faculdade de Ciências Médicas de São José dos Campos, São José dos Campos, SP, Brazil.; Universidade Estadual de Campinas Campinas SP Brazil Universidade Estadual de Campinas, Campinas, SP, Brazil.; Policlínicas Municipal de Sorocaba Sorocaba SP Brazil Policlínicas Municipal de Sorocaba, Sorocaba, SP, Brazil.; Universidade Federal do Rio Grande do Sul Porto Alegre RS Brazil Universidade Federal do Rio Grande do Sul, Porto Alegre, RS. Brazil.; Departamento de Obstetrícia Escola Paulista de Medicina São Paulo SP Brazil Departamento de Obstetrícia, Escola Paulista de Medicina, São Paulo, SP, Brazil.; Universidade de São Paulo Faculdade de Medicina, Hospital das Clínicas Instituto do Coração São Paulo SP Brazil Universidade de São Paulo, Faculdade de Medicina, Hospital das Clínicas, Instituto do Coração, Divisão de Pneumologia, São Paulo, SP, Brazil.; Universidade do Estado do Rio de Janeiro Faculdade de Ciências Médicas, Disciplina de Pneumologia e Tisiologia Rio de Janeiro RJ Brazil Universidade do Estado do Rio de Janeiro, Faculdade de Ciências Médicas, Disciplina de Pneumologia e Tisiologia, Rio de Janeiro, RJ, Brazil.; Universidade Federal de São Paulo Escola Paulista de Medicina São Paulo SP Brazil Universidade Federal de São Paulo, Escola Paulista de Medicina, São Paulo, SP, Brazil.

## Abstract

•Asthma is the most common lung disease during pregnancy and its diagnosis is determined in the same way in pregnant and non-pregnant women.

•Spirometry is a simple test used to confirm and monitor the disease, and has no contraindications for use during pregnancy both in the pre- and post-bronchodilator phase.

•The control of asthma before pregnancy is the main predictor of disease severity during pregnancy. Other predictors of asthma attacks include smoking, overweight and obesity.

•Inadvertent interruption of maintenance medication is one of the factors most associated with exacerbation and complications related to asthma during pregnancy.

•In general, treatment of pregnant women with asthma should be similar to that of non-pregnant women. Inhaled corticosteroids (ICS) are the main medication to achieve and maintain control of the disease during pregnancy.

•Corticosteroids prescribed for maintenance treatment of chronic asthma have no effect on accelerating fetal maturity. The usual protocol should be used when this acceleration is necessary.

•Moderate asthma exacerbation includes at least one of the following criteria: 1) worsening of respiratory symptoms; 2) worsening of lung function; 3) increased use of inhaled pump medication (e.g., salbutamol; at least two-day duration).

•Severe asthma exacerbation includes at least one of the following criteria: 1) use of systemic corticosteroids or increased dose of maintenance oral corticosteroids for at least three days; 2) hospitalization or visit to the emergency room (ER) due to asthma requiring the use of systemic corticosteroids.

•Asthma does not normally affect labor or the choice of delivery route.

**Recommendations**

- Ideally, all pregnant women with a history of asthma should be referred to high-risk pregnancy referral centers.
- Asthma control criteria should be monitored and assessed monthly (Table 1).
  **Table 1:** Asthma control criteria

Daytime symptoms > 2 times per week
Nocturnal awakenings due to asthma
Use of rescue medications > 2 times per week
Limitation of activities due to asthma

Source: Adapted from Reddel et al. (2022).^(1)^
- Maintenance asthma medications should not be inadvertently suspended early in pregnancy.
- The basis of maintenance drug treatment for asthma is the use of ICS with or without a long-acting β2-agonist (LABA).
- Treatment of asthma exacerbation includes the use of inhaled β2-agonist + anticholinergic, the use of systemic corticosteroids, and O_2_ supplementation if peripheral O_2_ saturation <95%. Blood glucose levels should be monitored due to the significant effects of hypoglycemia resulting from the use of β2-agonist bronchodilators.
- It is important to provide regular information about the importance of: 1) avoiding triggers (pollen, pet dander, dust, pollutants, exercise, climate changes, respiratory tract infections and smoking); 2) getting vaccinated annually against influenza; 3) adhering to medications.
- Hospitalization should be considered when a pregnant asthmatic patient does not respond to initial treatment with bronchodilators or if she presents with a severe or very severe exacerbation.
- Oxytocin is the drug of choice for inducing labor and controlling postpartum hemorrhage, since the use of prostaglandin analogues can cause bronchoconstriction.
- Morphine and meperidine should be avoided for controlling peripartum pain, as they can induce the release of histamines.
- Women who are currently receiving or have recently taken (within the previous four weeks) systemic corticosteroids should receive them at a stress dose (hydrocortisone 100 mg every eight hours) during labor and for 24 hours after delivery to prevent maternal adrenal crisis.
- In cases of acute asthma exacerbation, elective delivery may be postponed until the pregnant woman’s symptoms are relieved.
- Prednisone, theophylline, antihistamines, ICS, and β2-agonists are not contraindicated during breastfeeding, which should be encouraged.

## Background

The Global Initiative for Asthma (GINA) defines asthma as “a heterogeneous disease, generally characterized by chronic inflammation of the airways, with respiratory symptoms such as wheezing, shortness of breath, chest tightness and cough that vary over time and in intensity, together with variable limitation of expiratory airflow”.^(1)^ The female gender and low family income are also factors for higher prevalence.^(2)^ Asthma is the most common lung disease found during pregnancy, occurring in 3-8% of pregnant women.^(3-5)^ In the third trimester, increased respiratory drive can lead to similar symptoms and is not synonymous with the disease, but associated with pregnancy itself.^(6-8)^ In this document, we aim to explore in greater depth the aspects related to asthma and pregnancy that can contribute to the adequate care of pregnant women affected by this disease.

### How to diagnose asthma during pregnancy?

The diagnosis in pregnant women is determined in the same way as in non-pregnant women,^(7)^ based on the combination of compatible clinical history and demonstration of variable airflow obstruction. The characteristic respiratory symptoms of asthma are wheezing, coughing, retrosternal chest tightness and dyspnea. Data that reinforce the diagnosis are the presence of two or more symptoms described above, worsening at night and/or early in the morning, symptoms that vary in presence and intensity over time, and can be triggered by viral infection, exercise, exposure to allergens, sudden changes in temperature and exposure to strong odors, cigarettes and other pollutants. A previous pathological history of atopic dermatitis, allergic rhinitis, food allergy and/or other atopic comorbidities, as well as a positive family history of asthma and/or atopy also increase the likelihood of asthma.^(1,9)^ However, other data reduce it, such as isolated cough without any other respiratory symptoms, presence of stridor, chronic sputum production and typical chest pain.^(1)^

### Can we perform spirometry on pregnant women?

Spirometry is a simple test and has no contraindications during pregnancy, either in the pre- or post-bronchodilator phase. Although pregnancy is marked by physiological changes in the lungs, there is no change in the main spirometry parameters, such as forced vital capacity (FVC) and forced expiratory volume in one second (FEV1), nor in the FEV1/FVC ratio. Measurement of peak expiratory flow (PEF) can also be performed and, like spirometry,^(8,10)^ its performance in pregnant women can be important to help determine if the symptoms are due to asthma. The presence of obstructive respiratory disorder with a positive bronchodilator test or PEF variability supports the diagnosis of asthma.^(10,11)^

### Where can pregnant women with asthma receive antenatal care?

Ideally, all pregnant women with a history of asthma should be referred to high-risk pregnancy referral centers and monitored by a multidisciplinary team, as pregnancy is considered a risk factor for exacerbation of the condition. Their medications should be reviewed every three to six weeks, depending on the severity. In cases of severe or difficult-to-control asthma, referral and joint care with a pulmonologist at a tertiary center for women’s health care, possibly with hospitalization, are mandatory.^(12)^

### How does pregnancy affect asthma and how does asthma affect pregnancy?

The effects of pregnancy on asthma are quite variable. A significant proportion of women with asthma may experience worsening during pregnancy.^(13,14)^ In a prospective cohort, asthma worsened in 40% of women and remained the same in 60% of them. Contrary to previous studies, asthma did not appear to improve during pregnancy.^(14,15)^

Asthma control before pregnancy is the main predictor of disease severity during pregnancy, and may result in exacerbation in approximately 50% of cases.^(12)^Asthma attacks or exacerbations during pregnancy are more frequent in the second trimester of pregnancy. A possible explanation for this finding would be the inadvertent discontinuation of medication use by women as soon as they discover the pregnancy, which would culminate in exacerbations in the second trimester. Other predictors of asthma attacks during pregnancy include smoking, overweight and obesity.^(16-19)^

The severity of asthma and the need for hospitalization due to uncontrolled asthma are associated with adverse perinatal outcomes. Data indicate that children of asthmatic mothers have a higher risk of developing asthma in childhood.^(14)^

In general, women with asthma, especially those with well-controlled asthma and less severe disease, have good gestational outcomes. However, pregnant women with asthma may be at a higher risk of developing gestational complications, such as prematurity, spontaneous abortion, preeclampsia, fetal growth restriction, gestational diabetes, placental abruption, placenta previa, congenital anomalies, and cesarean delivery.^(20-24)^ Patients at higher risk of developing complications are those with suboptimal disease control. Inadequate disease control, greater severity or, in isolation, reduced FEV1, for example, are associated with hypertensive disorders and prematurity.^(25)^ Dysfunction in immunological adaptation during pregnancy and maternal hypoxemic episodes are the main mechanisms associated with the development of gestational complications. Thus, the main prevention of adverse outcomes is good asthma control and prevention of exacerbation episodes, also including continuous surveillance and multidisciplinary care.^(7)^

### How to assess asthma control and severity?

Asthma can be classified according to the level of clinical control, namely; controlled, partially controlled and uncontrolled, as established by GINA in 2022^(1)^ (Table 1).

Partially controlled asthma presents one or two criteria; uncontrolled asthma meets three or more control criteria;^(1,26)^ and controlled asthma does not present any of the criteria mentioned. Another way to classify asthma during pregnancy is according to its severity. Note that this classification is no longer used in favor of the classification made by control and step of treatment. In this classification, asthma can be considered mild, moderate or severe. This classification takes into account the history of the pregnant woman and above all, the amount and complexity of the treatment involved in maintaining good clinical control.

### How to treat asthma during pregnancy?

The aim of treatment is to achieve and maintain current control of the disease and prevent future risks (exacerbations, disease instability, accelerated loss of lung function, and adverse effects of treatment).^(27)^ Current guidelines are consistent in stating that the treatment of pregnant women with asthma should be similar to that of non-pregnant women, with inhaled corticosteroids (ICS) adopted as the main medication for achieving and maintaining control during pregnancy.^(1,9,28)^ The basis of pharmacological maintenance treatment of asthma is the use of ICS associated or not with a long-acting β2-agonist (LABA).^(1,9)^ LABAs associated with ICS are the most commonly used medications for asthma control and, compared to short-acting beta-2-agonists (SABAs), they provide more prolonged bronchodilation with greater reduction in symptoms, increased lung function, and less need for rescue SABAs. As the use of LABAs (formoterol or salmeterol) alone has been previously associated with increased asthma-related mortality in general, they are recommended only as controller therapy in combination with ICS. The use of budesonide + formoterol or beclomethasone + formoterol rescue is the preferred therapy in step 1 instead of SABA, since the combination of ICS + formoterol has been associated with a reduction in asthma exacerbations, in addition to improvements in PEF and FEV1, compared to the use of rescue SABA alone. This combination (budesonide or beclomethasone + formoterol, if necessary) may also be indicated instead of ICS alone in step 2 or associated with a fixed dose of ICS to treat moderate to severe asthma^(1,9,28)^ (Table 2).

**Table 2:** Medications for asthma during pregnancy and the puerperal period with breastfeeding

Category	Medication	Recommendation during pregnancy	Breastfeeding
Inhaled corticosteroid	Budesonide	Maintain use	Safe
Systemic corticosteroid		Recommended in moderate-severe crises Individual risk and benefit Avoid in the first trimester	Use with caution In the case of prednisone, avoid breastfeeding for 3-4 hours
Long-acting inhaled beta-adrenergic agonist	Formoterol Salmeterol	Maintain use Associate with inhaled corticosteroids	Maintain use
Short-acting beta-adrenergic agonist	Salbutamol Terbutaline	Use in times of crises Do not use as monotherapy	Safe
Short-acting muscarinic antagonist - inhaled	Ipratropium bromide	Individualized use Use in moderate-severe crises	Safe
Monoclonal antibodies	Omalizumab (anti-IgE)	No evidence of risks in animal studies Avoid starting use during pregnancy Maintain use with decision based on individual needs Avoid other monoclonal antibodies	Low concentration in breast milk. Individualized decision
Anti-leukotrienes	Montelukast	Avoid use Related to risk of prematurity, diabetes and maternal hypertension	Do not use

Source: Adapted from Vieira et al. (2022).^(12)^

The goal of asthma management in pregnant women with ICS or a combination of ICS + LABA is to ensure adequate control and prevent severe exacerbations, which are known to increase the risk of congenital anomalies. The ultra-LABAs (e.g., indacaterol and vilanterol) are LABAs more recently used in fixed daily combination with ICS. Although animal studies with ultra-LABAs suggest a low risk of congenital anomalies, there are no data available in humans. Therefore, these agents are not the first choice in pregnancy unless a fixed combination regimen once a day is needed to ensure adherence.^(28)^ As additional maintenance therapy, antileukotrienes, anticholinergic agents, theophylline and, more recently, biological therapies can also be used, taking into account the recommendations presented in table 2. Montelukast and zafirlukast, the latter not yet available in Brazil, are selective leukotriene receptor antagonists (LTRAs) indicated as maintenance controller therapies for asthma. Although data on the use of both agents are limited in pregnancy, montelukast is the first-line antileukotriene therapy during pregnancy because it has been more extensively studied.

Although theophylline was considered the mainstay of asthma medication because of its bronchodilator and anti-inflammatory activity, its use has declined in recent decades after the introduction of ICS and concerns about its toxicity and side-effect profile. Theophylline has been previously used without evidence of teratogenic effects, but is no longer recommended as first-line therapy and should only be recommended as an alternative therapeutic agent for additional control of moderate to severe asthma in exceptional circumstances.

Anticholinergic agents include the short-acting muscarinic antagonist (SAMA), ipratropium, and long-acting muscarinic antagonists (LAMAs), including tiotropium bromide, glycopyrronium bromide, and umeclidinium. Anticholinergics induce bronchodilation by inhibiting muscarinic receptors on smooth muscle. LAMAs are recommended as adjunctive therapy for moderate to severe persistent asthma, although no well-controlled clinical trials of tiotropium or the other LAMAs have been conducted specifically in pregnant women.

Oral corticosteroids (OCs) may be necessary for the treatment of acute exacerbations or used in low doses for the management of severe asthma, and are considered a last resort for maintenance treatment. More data on the safety of prednisone are available because it has been more extensively studied in pregnant women. All women should be informed that the benefits of OC treatment outweigh the risks if truly necessary, but they should also be warned about the adverse events with chronic use of OCs.^(29)^

**Figure 1:**
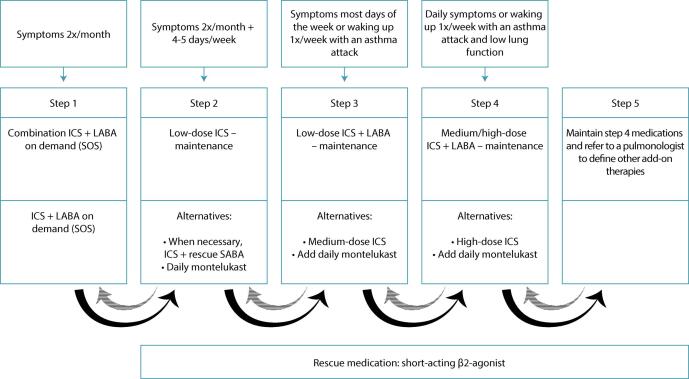
Proposed treatment flowcharts. The solid arrow shows the progression of medication use and the hatched arrow shows the de-escalation of medications

Although treatment adjustments can be made during pregnancy, given the evidence of adverse outcomes in pregnancy and childhood, the GINA recommends the monthly monitoring and assessment in asthma control when exacerbations occur during pregnancy^(1,30)^ (Figure 1).

### Is there concern about medications (beta-blockers, ophthalmic solutions, terbutaline, misoprostol, acetylsalicylic acid [ASA]) that interfere with the treatment/management of asthmatic patients?

Some medications can interfere with asthma control. Approximately 15-20% of asthmatic women are hypersensitive to ASA and other nonsteroidal anti-inflammatory drugs (ibuprofen, piroxicam, ketoprofen), so it is recommended to avoid these medications during pregnancy and the puerperal period. The use of analgesics such as morphine and meperidine can trigger asthma attacks by releasing histamines and should be avoided. The use of oral beta-blockers and beta-blocker eye drops can trigger bronchospasms and asthma exacerbation. Gastroesophageal reflux can worsen asthma, so the use of antacids and histamine receptor antagonists should be authorized. The need for medications can vary throughout pregnancy, as well as the severity of the condition. Generally, the need for medications is greater in the second trimester. Inhaled corticosteroids are safe and significantly help in controlling asthma and do not have any effects on fetal development. However, the systemic use of corticosteroid therapy should be avoided in the first trimester, as it may be related to the occurrence of low birth weight, prematurity, gestational hypertensive disorders and cleft palate, among others. It is important to remember that corticosteroids as maintenance treatment for chronic asthma do not have an effect on accelerating fetal lung maturity, and the usual protocol should be used when it is necessary to accelerate fetal lung maturity.^(31)^

### Non-pharmacological strategies

Education of both physicians and patients about the safety of controller medication is one of the most important non-pharmacological strategies, considering that a significant proportion of pregnant women with asthma stop or reduce their medications early in pregnancy. Pregnant women should be informed about the nature of the disease, the therapy used during pregnancy, complications, how to avoid triggers, appropriate use of devices, and the importance of adherence to therapy. Guidelines agree that avoiding triggers is an important component of asthma management. Stimuli such as pollen, pet dander, dust, exercise, climate change, emotions, upper respiratory tract infections, some medications that worsen asthma control, and smoking should be avoided, or at least reduced as much as possible. Asthmatic women of reproductive age and pregnant women should also be advised about the importance of annual influenza vaccination, as viral infections are frequent triggers for asthma exacerbations. Active and passive smoking should be avoided completely. The National Asthma Council (NAC) recommends adequate control of comorbidities such as allergic rhinitis and gastroesophageal reflux, which can mimic or aggravate asthma symptoms. In addition, stress and mental illnesses should also be controlled to prevent asthma exacerbations.^(30)^

### How to manage the use of immunobiologicals in pregnant women with severe asthma?

With the development of knowledge about the pathophysiology of the disease, different immunobiological agents have been used to treat patients with severe allergic and non-allergic eosinophilic asthma.^(32)^ However, data on their use during pregnancy are scarce.^(33)^

Immunobiologicals are indicated for patients in stage 5 of treatment, that is, with severe asthma, defined as that which remains uncontrolled with the maximum optimized treatment or requiring this treatment to prevent the disease from becoming uncontrolled, despite the suppression or minimization of worsening factors. Maximum treatment means the use of high doses of ICS (budesonide ≥ 800 µg or equivalent, according to GINA guidelines, or budesonide ≥ 1,600 µg or equivalent, according to the Brazilian Recommendations for the Management of Severe Asthma 2021 and the American Thoracic Society/European Respiratory Society consensus)^(34)^ and the need for a second controller medication in the previous year or the use of OC on ≥ 50% of the days in the previous year.^(1,32)^

To date, omalizumab is the only immunobiological for asthma with safety data during pregnancy. Although limited, these data are available in The Observational Study of the Use and Safety of Xolair (omalizumab) during Pregnancy Trial (EXPECT) registry.^(35)^ Omalizumab is a recombinant humanized anti-IgE monoclonal antibody and has been approved as an additive therapy for patients with moderate and severe allergic asthma unresponsive to high doses of ICS-LABA. It binds to the high-affinity receptor in the Fc region of IgE (FcεRI), preventing its binding to the receptor on the membrane of mast cells and basophils, actions that lead to reduced activation of these cells.^(36)^ Data from clinical studies show a reduction in both circulating free IgE levels and FcεRI receptors. It is one of the treatment options in step 5 of severe allergic asthma.

Two large observational studies – EXPECT and QECC – showed that the prevalence of major congenital anomalies was approximately 1 in 11 (8.1% vs. 8.9% in the EXPECT and QECC studies, respectively), the percentage of live births was similar in both groups (99.1% in EXPECT and 99.3% in QECC), and preterm and small-for-gestational-age births in EXPECT and QECC were identified in 15.0%-11.3% and 9.7%-15.8%, respectively. Therefore, there was no evidence of an increased risk of major congenital anomalies in the group of pregnant women exposed to omalizumab compared to unexposed pregnant women. However, as this is an observational study with methodological limitations, the absence of increased risk cannot be considered definitive.^(37)^

There are still no prospective data available in humans on the efficacy and safety of other monoclonal antibodies such as mepolizumab, reslizumab (not available in Brazil), benralizumab, dupilumab, and tezepelumab for the treatment of severe asthma during pregnancy, but some databases already collect information on these medications.^(28,38,39)^

### How to identify, classify, and treat asthma exacerbations?

Asthma exacerbations represent an acute worsening of symptoms and lung function in relation to the patient’s usual state, which requires a change in treatment. Exacerbations during pregnancy may result from mechanical or hormonal changes, as well as the reduction or cessation of control medications. The risk of exacerbation in pregnant women with asthma is 8% in mild asthma, 47% in moderate asthma, and 65% in severe asthma, and 20% of pregnant women with asthma have an exacerbation that requires medication intervention.^(40-43)^ Patients at higher risk of asthma-related death should be identified and flagged for more frequent evaluations, recognition, and early intervention to stabilize the condition.^(1,19)^

### Assessment of severity

Some data from the patient’s history may be associated with a higher risk of near-fatal or fatal progression of asthma exacerbation in adults, both in pregnant and non-pregnant women:

- Previous severe asthma attack requiring admission to an intensive care unit or mechanical ventilation;
- Hospitalization or ER visit due to asthma in the previous year;
- Frequent use of systemic corticosteroids (marker of severity);
- Use of two or more vials of metered-dose bronchodilator aerosol per month;
- Psychosocial problems (for example: anxiety, depression, obsessive-compulsive disorder, low socioeconomic status, difficulty in accessing care, lack of adherence to treatment and smoking);
- Irregular use of ICS;
- Labile asthma with marked variations in lung function (>30% of PEF or FEV1);
- Food allergy in asthmatic patient.

Therefore, the clinical history obtained during emergency care should be reviewed, seeking the probable cause of the current exacerbation and comorbidities.^(44)^

Objective data from the physical examination, such as respiratory rate, heart rate and presence or absence of bilateral wheezing and retraction of accessory muscles help in the classification of severity, as shown in table 3.

**Table 3:** Classification of asthma severity

	Mild	Moderate	Severe	Imminent CPA
**General**	Normal	Agitated	Agitated	Confused or drowsy
**Dyspnea**	With physical activity	Speaking	At rest	
**Body position**	Can lie down	Prefers to be seated	Cannot lie down	
**Fala**	Complete sentences	Incomplete sentences	Words	Cannot speak
**Respiratory rate**	Normal or increased	Increased	>30 rpm	
**Accessory muscles**	Does not usually use	Does not generally use	Uses	Paradoxical breathing
**Auscultation**	Moderate expiratory wheezing	Diffuse expiratory wheezing	Diffuse inspiratory and expiratory wheezing	No wheezing
**Heart rate**	<100 bpm	100-120 bpm	>120 bpm	Relative bradycardia
**Pulsus paradoxus**	<10 mmHg	10-25 mmHg	>25 mmHg	
**PaO_2_**	Normal	>60 mmHg	<60 mmHg	
**PaCO_2_**	<45 mmHg	<45 mmHg	>45 mmHg	
**PEF (predicted)**	>70%	50%-70%	≤50%	<30%
**SpO_2_**	>95%	91%-95%	<90%	

CPA: cardiopulmonary arrest; RPM: respiratory incursions per minute; BPM: beats per minute; PaO_2_: partial pressure of O_2_; PaCO_2_: partial pressure of CO_2_; PEF: peak expiratory flow; SpO_2_: peripheral O_2_ Source: Adapted from Pizzichini et al. (2020).^(9)^

The patient may have low perception of symptoms even in a severe and refractory crisis. Therefore, objective measures such as PEF measurement are essential. The patient should be monitored continuously during treatment, preferably with serial PEF measurements.

### Classification of asthma exacerbations

A moderate asthma exacerbation should include at least one of the following criteria:

- Worsening of respiratory symptoms;
- Worsening of lung function;
- Increased use of rescue medication.

The condition should last at least two days, but not being severe enough to require the use of systemic corticosteroids. An exacerbation that results in a visit to the ER, although without the use of systemic corticosteroids, should be considered as moderate.

Severe asthma exacerbation must include at least one of the following criteria:

- Use of systemic corticosteroids (tablets, suspensions or injections) or increased maintenance oral corticosteroid dose for at least three days (corticosteroid pulses separated by one week or more should be interpreted as separate events);
- Hospitalization or visit to the ER due to asthma, which requires the use of systemic corticosteroids.

*Additional tests during exacerbation*: reserved for classifying severity and assessing associated precipitating factors and complications:^(9)^

- Peripheral oxygen saturation (SpO_2_): the goal is to keep it ≥ 95%;
- Chest X-ray: if the response to initial treatment is not appropriate and if there is a possibility of complications such as atelectasis, pneumonia, pleural effusion, pneumothorax or pneumomediastinum, for example;
- Arterial blood gas analysis: if there is significant respiratory distress or hypoventilation, FEV1 or PEF < 30% of predicted;
- Complete blood count: if there is suspicion of infection (fever or cough with purulent sputum);
- Electrolytes: monitoring of complications due to the use of β2-agonists (hypokalemia) and in the coexistence of cardiovascular diseases and use of diuretics;
- Electrocardiogram: if there is heart disease or concomitant chronic obstructive pulmonary disease.

### Treatment of exacerbation

The aims of the treatment^(45)^ are to reverse airflow limitation, correct hypoxemia and reduce future recurrences.

### How to treat asthma exacerbation?

Treatment is similar to that of other non-asthmatic patients:

- Inhaled β2-agonist + anticholinergic;
- Systemic corticosteroid;
- O_2_ supplementation if SpO_2_ < 95%;
- Arterial blood gas analysis: observe partial pressure of CO_2_ (pCO_2)_.

The GINA recommends immediate and continuous treatment with SABA, oxygen therapy, and early administration of systemic corticosteroids. Blood glucose should be monitored because of the effects of hypoglycemia on the fetus. Neonatal hypoglycemia may be observed, especially in premature infants, when high doses of beta-agonists have been administered in the 48 hours before delivery. If high doses of SABA have been administered during labor and delivery, blood glucose levels should also be monitored in the newborn during the first 24 hours.^(1,46)^

*Short-acting* β*2-agonists (SABA):* These are the drugs of choice for relieving bronchospasm symptoms during acute exacerbations due to their rapid onset of action, and should be used with a spacer. The inhalation route is preferred, since parenteral use is associated with increased side effects without therapeutic gain. Parenteral use should be restricted to situations of imminent respiratory arrest. Salbutamol is the currently used SABA, available in a 100 mcg/dose aerosol for use with a spacer, or 10-20 drops diluted in 3-5 mL of 0.9% saline for nebulization. Three inhalations/nebulizations are recommended within the first hour of admission to the ER, reassessing the patient and the frequency of inhalations. If the aerosol is used with a spacer, 4-8 jets every 20 minutes are recommended in the first hour. The most common side effects are: tachycardia, palpitation, tremors, anxiety and hypokalemia.

The method of administration using a metered-dose inhaler with a spacer has proven as effective as nebulizers, in addition to being more economical and providing faster application of the desired dose. In addition, the use of nebulizers can disseminate aerosols and potentially contribute to the spread of respiratory infections.^(1)^

The use of medication via the parenteral route, available for terbutaline and salbutamol, is an exception and indicated only when the patient is unable to use the inhalation route, which is rare. The systemic route causes more side effects, without improvement in clinical or functional parameters, and is reserved for cases of severe bronchospasm and in the absence of response to inhalation measures.

*Short-acting muscarinic antagonist (SAMA):* The usefulness of anticholinergics in asthma attacks is not yet well defined. Meta-analyzes have shown benefits in relation to the use of ipratropium in reducing the hospitalization rate in the subgroup of patients with severe asthma exacerbation.

Ipratropium bromide can be used in the treatment of moderate to severe asthma exacerbations associated with SABA, resulting in fewer hospitalizations and a greater degree of improvement in lung function or in its replacement, as in the case of cardiac arrhythmia as a side effect (dose: 40 drops). The main side effects are dryness of the oral mucosa, glaucoma and urinary retention.

*Systemic glucocorticoids:* These are indicated in the treatment of exacerbations that do not respond well to initial treatment with bronchodilators. They promote faster resolution of airflow obstruction and reduce the recurrence rate. During the exacerbation, they should be administered as a pulse for patients on ICS treatment. Upon discharge from the emergency department and after a severe exacerbation, courses of five to 14 days should be prescribed (dose: 1-2 mg/kg/day, maximum 60 mg) without the need for weaning. The main side effects occur after prolonged use and/or high doses, including: high blood pressure, osteoporosis, changes in glucose metabolism, fluid retention, cushingoid facies, weight gain and aseptic necrosis of the femoral head.

The doses of systemic glucocorticoids are: prednisone – 1-2 mg/kg/day (40-60 mg) – or prednisolone; methylprednisolone – initial dose of 40-60 mg in the acute phase and, thereafter, every eight hours or every six hours (avoid doses greater than 180 mg/day); hydrocortisone: 200-300 mg intravenously and then 100-200 mg every six hours (avoid higher doses than 800 mg/day).

At the time of discharge from the ER, the ICS should be prescribed for patients with persistent asthma, associated with a course of oral corticosteroids. The patient should also be advised to seek regular outpatient treatment as soon as possible, preferably within a week and with a specialist.

*Methylxanthines*: There is no current evidence of benefit from their use in the treatment of acute asthma attacks. The use of methylxanthines is currently considered second-line treatment.

*Sedatives:* Their use should be judicious when there is no need for orotracheal intubation, as they can be a factor for apnea.

*Non-invasive ventilation (NIV) and invasive ventilation (IV):* Ventilatory support in acute asthma should be instituted with the aim to maintain adequate gas exchange while first-line medication is administered, reducing resistance and inflammation in the airways. Oxygen supplementation, NIV and IV are techniques available in this situation. Management is no different from that of non-pregnant women.

The goal of O_2_ supplementation is to achieve arterial oxygen saturation equal to or greater than 95% in order to ensure adequate oxygen supply to the tissues, including the respiratory muscles, preventing vasoconstriction and hypoxia induced by ventilation-perfusion disorders, which are present in severe attacks. The best way to provide oxygen to asthmatics undergoing spontaneous ventilation is through a low-flow nasal catheter (up to 2 L/min) or a mask with a Venturi device and controlled fraction of inspired O_2_ (FiO_2_).

Patients admitted with altered level of consciousness (agitation or drowsiness), bradycardia, or imminent cardiorespiratory arrest should immediately undergo ventilatory support with NIV or orotracheal intubation. Although NIV has shown efficacy in controlling patients with obstruction, it is not tolerated in many patients with asthma attacks, as it leads to high pressure peaks in the airways, causing discomfort to the patient. Non-invasive ventilation should be considered in cooperative and hemodynamically stable patients, and its use should not delay the indication for orotracheal intubation. Decreased level of consciousness, hemodynamic instability, and refractory hypoxemia are contraindications to NIV. Invasive ventilation should be indicated in patients with no improvement and signs of muscle fatigue, pH < 7.25, or PaCO_2_ > 55 mmHg.^(9)^ The use of medications and ventilatory support will depend on the ongoing clinical and laboratory evaluation of the patient, as shown in table 4.

**Table 4:** Algorithm for assessing response after initial treatment

Good: PEF > 70% No signs of severity	Prednisone 40 mg PO loading dose and prednisone 40 mg PO for 5 days + ICS (budesonide 400 to 800 mcg/day) and/or ICS + LABA + SABA depending on severity
ParTial: PEf 50%-70% Reduced severity	Maintain observation β2-agonist every 30-60 minutes for up to 4 hours Add ipratropium bromide and prednisone 40 mg PO
Absent or minor: PEF 35%-50% Persistent respiratory distress	Evaluate ARF/comorbidity β2-agonist every 20-30 minutes for up to 4 hours Add ipratropium bromide Systemic corticosteroid therapy Monitoring and never sedation Indication of ventilatory support, if necessary
Worse: PFE < 35% or not measurable Worse severity	Intensive care Evaluate ARF/comorbidity ARF monitoring Bronchodilator and parenteral corticosteroid

PEF: peak expiratory flow; PO: oral route; IC: inhaled corticosteroid; LABA: long-acting ß2-agonist; SABA: short-acting ß2-agonist bronchodilator; ARF: acute respiratory failure; ER: emergency room

### When to hospitalize?

Hospitalization should be indicated when the patient does not respond to initial treatment measures or if she initially presents with severe or very severe exacerbation.^(1)^

### Delivery route in asthmatic patients: are changes in delivery care needed?

Asthma does not normally affect labor nor the choice of delivery route. However, some points are relevant:^(47)^

- In labor induction, as well as for controlling postpartum hemorrhage, oxytocin is the drug of choice. If the use of prostaglandin analogues is necessary, prostaglandin E2 (dinoprostone) and prostaglandin E1 (misoprostol) are the safest given their bronchodilator effects. Prostaglandin F2-alpha (e.g., carboprost, which is not yet widely available in Brazil) can cause bronchoconstriction and should not be used to induce labor or control uterine hemorrhage.^(47,48)^
- Morphine and meperidine should be avoided to control peripartum pain, as they can induce the release of histamines, even though evidence regarding the relationship between cases of acute bronchospasm caused by these agents is lacking. Epidural analgesia is the best choice for pain control, as it reduces oxygen consumption and minute ventilation in the first and second stages of labor and generally provides an adequate anesthetic route if cesarean section is necessary. When general anesthesia is necessary, ketamine and halogenated anesthetics are preferable because of their bronchodilator effect.^(7,47)^
- Women who are currently receiving or have recently taken (in the last four weeks) systemic corticosteroids should receive stress-dose corticosteroids (e.g., hydrocortisone 100 mg every eight hours) during labor and for 24 hours after delivery to prevent maternal adrenal crises.^(7,47,49)^
- In patients with asthmatic manifestations, a pulse oximeter should be used to monitor SpO_2_.^(7,49)^
- In cases of acute exacerbation, elective delivery may be postponed until the pregnant woman’s symptoms are relieved.^(7)^

### Postpartum/puerperal period management in asthmatic patients

Care should be similar to that during pregnancy, and medications in use should not be discontinued until further clinical evaluation.

If the pregnant woman received high doses of SABA during labor and delivery, the newborn’s glucose levels should be monitored (especially in the case of premature babies) during the first 24 hours to diagnose possible hypoglycemia.^(7,49)^

Prednisone, theophylline, antihistamines, inhaled corticosteroids and β2-agonists are not contraindicated during breastfeeding, which should be encouraged.^(7,49)^

### Flow of care for pregnant women with asthma

**Figure 2:**
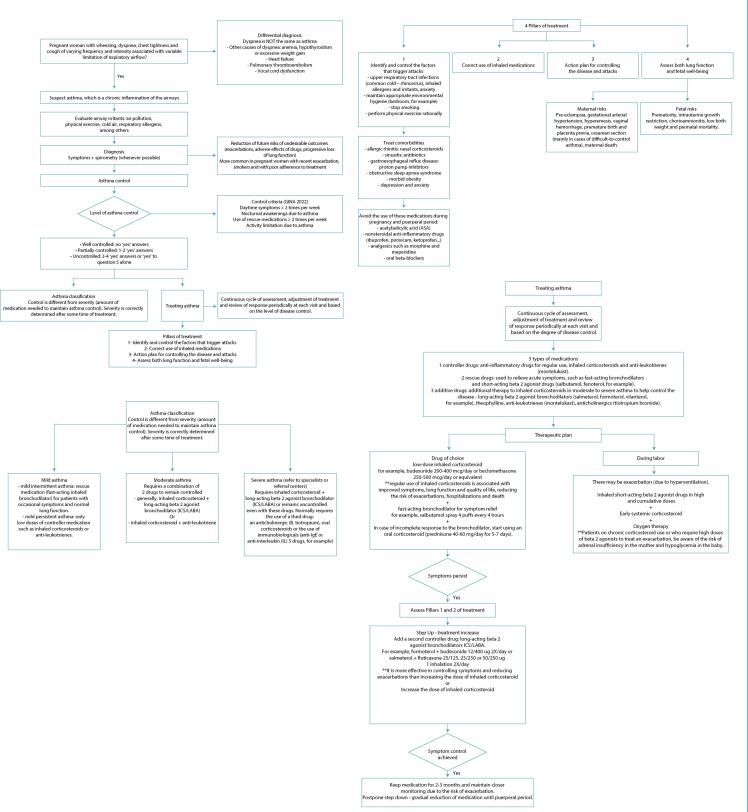
Flowchart of care (diagnosis and management) for pregnant women with asthma

We present the flows of care for pregnant women with asthma, including diagnosis and management (Figure 2).

## Final considerations

Adequate asthma control in the pre-gestational period and regular use of medications are essential steps to reduce asthma-related complications during pregnancy. Thus, the main prevention of adverse outcomes is good asthma control and prevention of exacerbation episodes, also including continuous monitoring and multidisciplinary care. The asthmatic patient should be monitored jointly by an obstetrician and a pulmonologist during antenatal care, as well as referred for high-risk antenatal care. The physician should be emphatic in advising the patient to avoid triggers and on the importance of adherence to treatment and not inadvertently stopping medication, which is crucial for the most favorable evolution of the pregnancy. Asthma control criteria should be performed monthly or if there are signs of change in the control pattern, and exacerbations should be promptly recognized and managed to avoid complications. De-escalation of medications, when applicable, should be encouraged to occur only after delivery.
